# Dabigatran – a case history demonstrating the need for comprehensive approaches to optimize the use of new drugs

**DOI:** 10.3389/fphar.2013.00039

**Published:** 2013-05-14

**Authors:** Rickard E. Malmström, Brian B. Godman, Eduard Diogene, Christoph Baumgärtel, Marion Bennie, Iain Bishop, Anna Brzezinska, Anna Bucsics, Stephen Campbell, Alessandra Ferrario, Alexander E. Finlayson, Jurij Fürst, Kristina Garuoliene, Miguel Gomes, Iñaki Gutiérrez-Ibarluzea, Alan Haycox, Krystyna Hviding, Harald Herholz, Mikael Hoffmann, Saira Jan, Jan Jones, Roberta Joppi, Marija Kalaba, Christina Kvalheim, Ott Laius, Irene Langner, Julie Lonsdale, Sven-Äke Lööv, Kamila Malinowska, Laura McCullagh, Ken Paterson, Vanda Markovic-Pekovic, Andrew Martin, Jutta Piessnegger, Gisbert Selke, Catherine Sermet, Steven Simoens, Cankat Tulunay, Dominik Tomek, Luka Vončina, Vera Vlahovic-Palcevski, Janet Wale, Michael Wilcock, Magdalena Wladysiuk, Menno van Woerkom, Corrine Zara, Lars L. Gustafsson

**Affiliations:** ^1^Clinical Pharmacology Unit, Department of Medicine, Karolinska Institutet, Karolinska University Hospital SolnaStockholm, Sweden; ^2^Division of Clinical Pharmacology, Department of Laboratory Medicine, Karolinska Institutet, Karolinska University Hospital HuddingeStockholm, Sweden; ^3^Liverpool Health Economics Centre, University of LiverpoolLiverpool, UK; ^4^Strathclyde Institute for Pharmacy and Biomedical Sciences, University of StrathclydeGlasgow, UK; ^5^Unitat de Coordinació i Estratégia del Medicament, Direcció Adjunta d’Afers Assistencials, Catalan Institute of HealthBarcelona, Spain; ^6^Austrian Medicines and Medical Devices AgencyWien, Austria; ^7^Information Services Division, NHS National Services ScotlandEdinburgh, UK; ^8^Agency for Health Technology AssessmentWarsaw, Poland; ^9^Hauptverband der Österreichischen SozialversicherungsträgerWien, Austria; ^10^Centre for Primary Care, Institute of Population Health, University of ManchesterManchester, UK; ^11^London School of Economics and Political Sciences, LSE HealthLondon, UK; ^12^King’s Centre for Global Health, Global Health Offices, Weston Education CentreLondon, UK; ^13^Health Insurance InstituteLjubljana, Slovenia; ^14^Medicines Reimbursement Department, National Health Insurance FundVilnius, Lithuania; ^15^Instituto Nacional da Farmácia e do MedicamentoLisboa, Portugal; ^16^Osteba Basque Office for Health Technology Assessment, Ministry of Health of the Basque CountryDonostia-San Sebastian, Vitoria-Gasteiz, Basque Country, Spain; ^17^Norwegian Medicines AgencyOslo, Norway; ^18^Kassenärztliche Vereinigung HessenFrankfurt am Main, Germany; ^19^Nätverk för läkemedelsepidemiologi, Department of Health Analysis, University HospitalLinköping, Sweden; ^20^Clinical Programs, Pharmacy Management, Horizon Blue Cross Blue Shield of New JerseyNewark, USA; ^21^Ninewells Hospital, NHS TaysideDundee, UK; ^22^Pharmaceutical Department, Local Health Unit of VeronaVerona, Italy; ^23^Republic Institute for Health InsuranceBelgrade, Serbia; ^24^State Agency of MedicinesTartu, Estonia; ^25^Wissenschaftliches Institut der AOKBerlin, Germany; ^26^Lancashire Commissioning Support Unit, Jubilee HouseLeyland, Lancashire, UK; ^27^Department of Healthcare Development, Stockholm County CouncilStockholm, Sweden; ^28^HTA ConsultingCracow, Poland; ^29^Public Health School, The Medical Centre of Postgraduate EducationWarsaw, Poland; ^30^National Centre for Pharmacoeconomics, St James’s HospitalDublin, Ireland; ^31^Scottish Medicines ConsortiumGlasgow, UK; ^32^Faculty of Medicine, University of Banja LukaBanja Luka, Bosnia and Herzegovina, Republic of Srpska; ^33^Ministry of Health and Social WelfareBanja Luka, Bosnia and Herzegovina, Republic of Srpska; ^34^NHS BuryBury, UK; ^35^Institut de Recherche et Documentation en Économie de la SantéParis, France; ^36^KU Leuven Department of Pharmaceutical and Pharmacological SciencesLeuven, Belgium; ^37^President of the Turkish Rational Drug Use PlatformAnkara, Turkey; ^38^Faculty of Pharmacy, Comenius UniversityBratislava, Slovakia; ^39^Faculty of Medicine, Slovak Medical UniversityBratislava, Slovakia; ^40^Ministry of HealthZagreb, Republic of Croatia; ^41^Unit for Clinical Pharmacology, University Hospital RijekaRijeka, Croatia; ^42^Independent Consumer AdvocateBrunswick, VIC, Australia; ^43^Prescribing Support Unit, c/o Pharmacy Department, Royal Cornwall Hospitals NHS TrustTruro, Cornwall, UK; ^44^Dutch Institute for Rational Use of MedicineUtrecht, Netherlands; ^45^Barcelona Health Region, Catalan Health ServiceBarcelona, Spain

**Keywords:** critical drug evaluation, dabigatran, demand-side measures, drug and therapeutics committees, managed introduction new medicines, pharmacovigilance, registries, risk sharing

## Abstract

**Background:** There are potential conflicts between authorities and companies to fund new premium priced drugs especially where there are safety and/or budget concerns. Dabigatran, a new oral anticoagulant for the prevention of stroke in patients with non-valvular atrial fibrillation (AF), exemplifies this issue. Whilst new effective treatments are needed, there are issues in the elderly with dabigatran due to variable drug concentrations, no known antidote and dependence on renal elimination. Published studies have shown dabigatran to be cost-effective but there are budget concerns given the prevalence of AF. There are also issues with potentially re-designing anticoagulant services. This has resulted in activities across countries to better manage its use.

**Objective:** To (i) review authority activities in over 30 countries and regions, (ii) use the findings to develop new models to better manage the entry of new drugs, and (iii) review the implications for all major stakeholder groups.

**Methodology:** Descriptive review and appraisal of activities regarding dabigatran and the development of guidance for groups through an iterative process.

**Results:** There has been a plethora of activities among authorities to manage the prescribing of dabigatran including extensive pre-launch activities, risk sharing arrangements, prescribing restrictions, and monitoring of prescribing post-launch. Reimbursement has been denied in some countries due to concerns with its budget impact and/or excessive bleeding. Development of a new model and future guidance is proposed to better manage the entry of new drugs, centering on three pillars of pre-, peri-, and post-launch activities.

**Conclusion:** Models for introducing new drugs are essential to optimize their prescribing especially where there are concerns. Without such models, new drugs may be withdrawn prematurely and/or struggle for funding.

## BACKGROUND

New medicines are of real value to patients when they improve their health either because they are more effective, have less side-effects, or are easier to administer than current standards. European health authorities also wish new drugs to be cost-effective (Garattini et al., 2008; Godman et al., 2008, 2009c, 2012d; Wettermark et al., 2008, 2010a; Coma et al., 2009; Sermet et al., 2010; Garuoliene et al., 2011b; Voncina and Strizrep, 2011; Vončina et al., 2011; Cheema et al., 2012; Markovic-Pekovic et al., 2012). Continued pressure on resources is already resulting in some countries unable to fund new premium priced drugs (Garuoliene et al., 2011a,b; Godman et al., 2011c, 2012b; Taylor, 2011), with the number of countries likely to increase with new drugs now being launched at US$300,000 (€228,000) per patient per year or more (Kaiser, 2012). Premium prices are of concern among authorities struggling to maintain, and potentially incompatible with, the European ideals of comprehensive and equitable healthcare (Garattini et al., 2008; Adamski et al., 2010; Godman et al., 2012b).

This may result in conflicts between authorities and pharmaceutical companies with the latter keen to re-coup the considerable monies spent on research and development as soon as possible through rapid reimbursement (DiMasi and Grabowski, 2007; Abraham, 2008; Jaroslawski and Toumi, 2011; Persson et al., 2012) as well as maintain profitability with established products (Shuchman, 2006; Abraham, 2008; Godman et al., 2010, 2011b,b; Vončina et al., 2011; Baumgärtel et al., 2012; Jackevicius et al., 2012). However, this can be at odds with the aims of health authorities and health insurance companies struggling to meet European ideals within available resources (Shuchman, 2006; Abraham, 2008; Garattini et al., 2008; Godman et al., 2010, 2011b, 2012b; Sermet et al., 2010; Garuoliene et al., 2011b; Voncina and Strizrep, 2011; Vončina et al., 2011; Baumgärtel et al., 2012; Ozierański et al., 2012). Marketing activities are seen as important by companies to achieve their aims in an increasingly competitive environment (Civaner, 2012); but these can involve considerable spending. Published studies suggest marketing costs can be as high as one-third of a company’s income (Civaner, 2012), with companies spending US$53bn (€40.2) in the US alone in 2004 marketing to physicians (Lexchin and Kohler, 2011; Godman et al., 2012b; Godman and Gustafsson, 2013). In addition, there have been concerns with aggressive lobbying and other indirect strategies by some companies (Mello et al., 2012; Ozierański et al., 2012), as well as with some of the marketing (Department of Justice, 2010; Griffin and Segal, 2010; Lexchin and Kohler, 2011; Fisk et al., 2012; Davies and Abraham, 2013) and other activities (Jackevicius et al., 2012; Baumgärtel et al., 2012; Ozierański et al., 2012; Davies and Abraham, 2013) to achieve their aims. This is despite the imposition of multi-million dollar fines (Davies and Abraham, 2013).

These conflicts can be greater when there are safety concerns with new drugs, and they are subsequently prescribed in a wider population than studied in randomized clinical trials. Typically Phase III clinical trials are conducted under ideal and highly controlled conditions to seek high internal validity to maximize the chance of demonstrating clinical benefit (Fritz and Cleland, 2003). However, this may lead to substantial differences from their subsequent use in clinical practice. Typically Phase III clinical trials do not include treatment preferences and/or multimodal treatment programs (Wells, 1999; Guthrie, 2000; Fritz and Cleland, 2003). Phase III clinical trials may also include a placebo group as a comparator in order to isolate the effects of a particular intervention (Fritz and Cleland, 2003). These situations can lead to concerns with the generalizability of the findings when new drugs are being considered as an alternative to current treatments, especially once prescribed in patients with greater co-morbidities than those enrolled into Phase III clinical trials.

For example, both cerivastatin and mibefradil had favorable benefit–risk profiles at market authorization, but their use in clinical practice, coupled with physicians ignoring recommended guidance, caused their withdrawal from the market (Friedman et al., 1999; Eichler et al., 2011). Previously in the 1980s zimelidine, the first selective serotonin re-uptake inhibitor, was withdrawn from the market due to hypersensitivity reactions and febrile reactions in connection to liver function disturbances, which later evolved into Guillain–Barré syndrome (GBS; Carlson, 1999, 2000). This withdrawal might have been avoided if zimelidine had been introduced in a stepwise fashion, as there was an average increase of GBS-risk of 25-fold among patients receiving zimelidine compared with the natural incidence of the disorder (Fagius et al., 1985).

Rofecoxib was also withdrawn following growing evidence of increased cardiovascular events such as heart attacks and stroke with long-term treatment (Merck, 2004; MHRA UK, 2004). Rofecoxib was seen as the most selective COX-II inhibitor among the first generation of this class with minimal COX-I activity (Davies and Jamali, 2004). Whilst this reduces gastrointestinal (GI) side-effects, this also reduced the cardioprotective effect of COX-I inhibitors that is similar to low-dose aspirin (Davies and Jamali, 2004; Bresalier et al., 2005). This protective effect of COX-I inhibitors led to a reduction in the risk of thrombotic cardiovascular events in patients treated with naproxen compared to rofecoxib (Reicin et al., 2001; Weir et al., 2003; Davies and Jamali, 2004), documented in the VIGOR study (Bombardier et al., 2000). The study specifically excluded patients who were taking concomitant aspirin or other antiplatelet drugs such as those with a recent history of myocardial infarction or stroke (Bombardier et al., 2000; Merck, 2002). The findings led to a caution being added to the product label in May 2002 in patients with a medical history of ischemic heart disease (FDA, 2002; Merck, 2002). The concerns with increased cardiovascular events associated with long-term rofecoxib therapy also led to the instigation of the APPROVe study (Bresalier et al., 2005). The findings of increased cardiovascular risk with rofecoxib (Bresalier et al., 2005) subsequently led to its withdrawal (Davies and Jamali, 2004; Merck, 2004; MHRA UK, 2004). There are ongoing debates whether the withdrawal of rofecoxib may have been avoided if there had not been appreciable marketing activities, including considerable direct to consumer advertising in the US, promoting the safety of COX-II inhibitors (Calfee and Pinell, 2005).

Natalizumab was withdrawn soon after its launch despite improved effectiveness in patients with relapsing multiple sclerosis. This was due to the development of progressive multifocal leukoencephalopathy (PML) in some patients (Kappos et al., 2011; Keegan, 2011). However, it was re-launched some 2 years later in Europe, but under strict prescribing regulations and with the instigation of research programs to clarify the benefit:risk ratios (Kappos et al., 2011; Keegan, 2011). More recently, rimonabant has been withdrawn from the market. Patients prescribed rimonabant experienced a higher incidence of anxiety, depression, and insomnia (Moreira et al., 2009; O’Shaughnessy, 2009; Ioannides-Demos et al., 2011). This led to advice that patients prescribed rimonabant should be investigated first for psychiatric illness and that rimonabant should not be prescribed in patients with mental illness (Moreira et al., 2009; O’Shaughnessy, 2009; Ioannides-Demos et al., 2011). However, this advice was sometimes ignored leading to its withdrawal due to increased risk of depression and suicidal ideation (European Medicines Agency [EMA], 2009; Godman et al., 2009c; Dietrich and Horvath, 2012; Wong et al., 2012). It may be that greater knowledge of the role of the hypothalamus in enabling the central nervous system to adapt to the changing environment could facilitate the discovery of new agents that are more effective and have a more acceptable benefit–risk profile (Wong et al., 2012). However, this remains to be seen.

New oral anticoagulants (NOACs) illustrate some of these tensions as they show promise in the prevention of stroke in patients with atrial fibrillation (AF), offering an alternative to warfarin without the need for INR (International Normalized Ratio) monitoring (Baetz and Spinler, 2008; Connolly et al., 2009; Malmström, 2009; Pink et al., 2011; Scottish Medicines Consortium, 2011; Banerjee et al., 2012; Godman et al., 2012d; Kansal et al., 2012; Mannuci et al., 2012; National Institute for Health and Clinical Excellence, 2012; Davidson et al., 2013; Joppi et al., 2013; Marshall et al., 2013; Rodriguez et al., 2013). This is in addition to venous thromboembolism prophylaxis for patients undergoing hip and knee surgery, and in the treatment of acute deep vein thrombosis and pulmonary embolism (Marshall et al., 2013). However, there are safety concerns especially in the elderly (Malmström, 2009; Pink et al., 2011; Godman et al., 2012d; Mannuci et al., 2012; Stollberger and Finsterer, 2013) in addition to potential compliance (Marshall et al., 2013; Rodriguez et al., 2013) and storage issues (Stollberger and Finsterer, 2013).

Atrial fibrillation is the most common clinically significant cardiac arrhythmia with an estimated prevalence of 1–2% of the population (Marshall et al., 2013). One in four adults over the age of 40 is likely to develop AF in their lifetime, higher in those aged over 80 (Lloyd-Jones et al., 2004; Stewart et al., 2004; Camm et al., 2010; Pink et al., 2011; Mannuci et al., 2012; Davidson et al., 2013). Current estimates suggest there are 4.5 million people in Europe with AF and 3.03 million in the US (Marshall et al., 2013), with the prevalence of AF likely to double in the next 50 years with ageing populations (Go et al., 2001; Lloyd-Jones et al., 2004; Luengo-Fernandez et al., 2006; Kirchhof et al., 2007; Connolly et al., 2009; Camm et al., 2010; Pink et al., 2011; Marshall et al., 2013). New drugs are needed since patients with AF have a fivefold increased risk of cardioembolic stroke compared with those in sinus rhythm (Stewart et al., 2004; Camm et al., 2010; Pink et al., 2011), with a cardioembolic stroke resulting in approximately 20% of patients dying in the acute phase and 60% developing severe disability (Mannuci et al., 2012). Incurred costs also tend to be higher in stroke patients with AF, with those patients who survive left more disabled by their stroke and more likely to have a recurrence than those with other causes of stroke (Luengo-Fernandez et al., 2006; Camm et al., 2010; Kansal et al., 2012). Initial incurred secondary care costs averaged GB£9667/patient (2005 costs) in patients with AF compared with an average of GB£5824 in other stroke patients (Luengo-Fernandez et al., 2006). As a consequence, the risk of death from AF related strokes is doubled compared with other forms of stroke, and the overall cost of care increased 1.5-fold (Kirchhof et al., 2007; Camm et al., 2010; Scottish Medicines Consortium, 2011; National Institute for Health and Clinical Excellence, 2012). Anticoagulant therapy with vitamin K antagonists (VKAs) can reduce by at least 60% the risk of stroke (Camm et al., 2010; Mannuci et al., 2012). However, there are concerns with warfarin due to the potential of bleeding, the need to tailor doses to the individual with too high a dose potentially causing serious complications and too low a dose losing protection, and the difficulties with maintaining some patients within INRs (Pink et al., 2011; Mannuci et al., 2012; Marshall et al., 2013).

Dabigatran received EU marketing authorization in August 2011 (Boehringer Ingelheim, 2011a; Marshall et al., 2013) for the prevention of stroke and systemic embolism/clot formation in adult patients with non-valvular AF with one or more of the following risk factors:

• Previous stroke, transient ischemic attack, or systemic embolism/clot formation
• Left ventricular ejection fraction <40%
• Symptomatic heart failure > New York Heart Association (NYHA) Class 2
• Age > 75 years
• Age > 65 years in combination with additional vascular risk, i.e., patients with diabetes mellitus, coronary artery disease, or arterial hypertension

Published studies showed a 9% reduction in the prevention of stroke or systemic embolism with dabigatran 110 mg twice daily and 34% for the 150 mg twice daily (Horsley, 2010; Mannuci et al., 2012; Davidson et al., 2013; Marshall et al., 2013). Overall mortality was also reduced by 12% for the highest dose of dabigatran, which reached statistical significance (Horsley, 2010; Mannuci et al., 2012). There was also an appreciable and consistent reduction in the risk of hemorrhagic stroke ranging from 69 to 74% depending on the dose of dabigatran (Horsley, 2010; Pink et al., 2011; Mannuci et al., 2012), with the 150 mg twice daily dose of dabigatran also providing a statistical significant reduction in ischemic stroke (24% risk reduction; Horsley, 2010; Pink et al., 2011; Mannuci et al., 2012). Dabigatran could also potentially require no monitoring compared with warfarin (Pink et al., 2011; Godman et al., 2012d; Mannuci et al., 2012; Marshall et al., 2013). As a result, dabigatran has the potential to be an important new treatment, especially where regular monitoring with warfarin is problematic or where there are adverse events or other patient issues with warfarin.

These improvements, coupled with potential savings with dabigatran with the opportunity to reduce patient monitoring, resulted in incremental cost-effectiveness ratios (ICERs) of GB£4831 (€5560)/quality adjusted life year (QALY) in patients under 80 versus warfarin and GB£7090 (€8150) above 80 (Kansal et al., 2012). A similar study in Sweden estimated the cost/QALY gained for dabigatran versus warfarin as €7742, increasing to €12,449 in patients who were well controlled with warfarin (Davidson et al., 2013). Other authors have published higher ICERs, i.e., GB£23,082 (€26,700)/QALY for high dose dabigatran versus warfarin (Pink et al., 2011; Scottish Medicines Consortium, 2011; Marshall et al., 2013). The manufacturer’s submission to the Scottish Medicines Consortium (SMC) suggested a cost/QALY of GB£6986 (€8030) versus warfarin. This estimate was based on the sequencing of dabigatran, starting with 150 mg twice daily for patients under the age of 80 who were subsequently switched to 110 mg twice daily when they reached 80 years (Scottish Medicines Consortium, 2011). The ICER increased to GB£13,347 (€15,350) when the model was adjusted to lower the potential savings from reduced INR monitoring to a more appropriate figure (Scottish Medicines Consortium, 2011; Marshall et al., 2013). The Evidence Review Group (ERG) of the National Institute for Health and Clinical Excellence (NICE) also had concerns with the model provided by the manufacturer and the cost of anticoagulation therapy (National Institute for Health and Clinical Excellence, 2012; Marshall et al., 2013). Under different assumptions, the ERG believed the base case ICER for dabigatran 150 mg twice daily increased from GB£6264 (€7200) to GB£24,173–29,131 (€27,790–33,490)/QALY (National Institute for Health and Clinical Excellence, 2012). This was due to two main weaknesses in the submitted model (National Institute for Health and Clinical Excellence, 2012). These included the lack of any potential switching of treatment from dabigatran back to warfarin as well as an overstatement of the costs of monitoring patients prescribed warfarin in practice (National Institute for Health and Clinical Excellence, 2012). There were also concerns that patient heterogeneity would be greater in practice than allowed for in the submitted models (National Institute for Health and Clinical Excellence, 2012; Marshall et al., 2013). However, both organizations recommended dabigatran as an alternative to warfarin in patients who meet the criteria outlined in the marketing authorization (Scottish Medicines Consortium, 2011; National Institute for Health and Clinical Excellence, 2012; Marshall et al., 2013), with NICE also recommending that dabigatran should only be prescribed after an informed discussion between clinicians and patients (National Institute for Health and Clinical Excellence, 2012). The National Centre for Pharmacoeconomics (NCPE) in Ireland recently concluded that the ICER for dabigatran versus warfarin was €6311/QALY in patients under 80 years and €20,654/QALY in patients 80 years or older. Extracranial hemorrhage was an important cost driver (versus warfarin in those 80 years and over) and disability costs were important across all comparisons (National Centre for Pharmacoeconomics, 2011).

However, there have been concerns with the rapid introduction of dabigatran, which led to an appreciable number of serious adverse events with the first 12 weeks of availability in the US (Institute for Safe Medication Practices, 2011). These were principally serious bleeding events or blood clots in the elderly (Institute for Safe Medication Practices, 2011). These concerns and others led the FDA to explore correlating reductions in stroke events with increasing plasma correlations alongside bleeding event rates (Thompson, 2010), with the guidance available when dabigatran was launched in Europe. In addition, re-examining and comparing the bleeding rates with warfarin and dabigatran (Southworth et al., 2013). These concerns have arisen due to dabigatran’s low mean oral bioavailability, considerable variation in plasma drug concentrations, and the complete dependence on renal elimination of the active metabolite (Stangier et al., 2008; Stangier and Clemens, 2009; Thompson, 2010; Liesenfeld et al., 2011; Douxfils et al., 2012; Huisman et al., 2012; Mannuci et al., 2012; Ten Cate, 2012). Consequently, any accumulation of dabigatran in patients with reduced renal function will increase their risk of excessive bleeding (Malmström, 2009; Legrand et al., 2011; Pink et al., 2011; Garber et al., 2012; Godman et al., 2012d; Harper et al., 2012; Huisman et al., 2012; Mannuci et al., 2012; Ten Cate, 2012), complicated by no known antidote (Rolfe et al., 2010; Institute for Safe Medication Practices, 2011; Pink et al., 2011; Godman et al., 2012d; Huisman et al., 2012; Mannuci et al., 2012; Marshall et al., 2013). This is important in this situation as patients in clinical practice are likely to be more elderly, have greater co-morbidities, and have reduced hepatic and renal functions, compared to the patients in the clinical trials (Joppi et al., 2013). There are also concerns with its budget impact given the growing prevalence of AF (Pink et al., 2011; Godman et al., 2012d; Midlands Therapeutic Review and Advisory Committee [MTRAC], 2012; Marshall et al., 2013). A number of health authorities across Europe have recognized these issues and initiated extensive pre- and peri-launch programs to educate physicians and the public regarding the optimal use of dabigatran, especially in elderly patients with poor renal function.

The principal objective of this paper is to review health authority and health insurance company activities across Europe pre-launch to post-launch of dabigatran for the prevention of stroke as an exemplar for developing future models to better manage the entry of new premium priced drugs. Subsequently, to use these strategies to suggest future activities that all key stakeholder groups could undertake to reduce the likelihood of new drugs being removed from the market place where there are concerns with their use in a wider patient population. Finally, to suggest activities that better manage expenditure on new drugs where there are concerns with their budget impact. This is important as concerns with the budget impact of new drugs are growing. This especially given the number of new drugs in development including new biological drugs (EvaluatePharma, 2012; Godman, 2013), which are now costing up to US$10,000–25,000 (€7580–18,960) per patient per month (Selyukh, 2011; Yukhananov, 2011; Godman et al., 2012b; Kaiser, 2012; UK Medicines Information, 2012; UKMi Medicines Information, 2013). This potentially inhibits the ability of governments to continue to provide equitable and comprehensive healthcare within current budgets.

## METHODOLOGY

A descriptive review of national, regional or local health authority, health insurance company or physician association activities across Europe regarding dabigatran up to and including the beginning of 2013 was conducted by one of the co-authors (Brian B. Godman). This was undertaken by collating and appraising relevant published papers and internal documents known to the co-authors as well as any pertinent documents available on the internet. Direct feedback was provided by the co-authors where there was limited or no data available in a particular country. The information provided by the co-authors was subsequently re-checked (Brian B. Godman) to enhance its accuracy. In total, information was collected from over 30 European countries and regions. We have used this methodological approach in previous publications involving health authority and health insurance company personnel when there has been a paucity of published data (Cheema et al., 2012; Godman et al., 2011b,c, 2010, 2012a,b; Adamski et al., 2010; Baumgärtel et al., 2012). The countries were chosen to provide differences in geography, epidemiology, financing of healthcare, available resources for healthcare as well as different approaches to the pricing and reimbursement of new drugs (Godman et al., 2008, 2010, 2011b, 2012d; Wettermark et al., 2008, 2010a; Coma et al., 2009; Sermet et al., 2010; Garuoliene et al., 2011a,b; Voncina and Strizrep, 2011; Cheema et al., 2012; Markovic-Pekovic et al., 2012; Godman and Gustafsson, 2013). This included both national and regional authorities in some countries, recognizing ongoing budget devolution, e.g., England, Scotland, Spain, and Sweden, as well as differences with the organization and funding of anticoagulant services.

Demand-side initiatives and reforms were collated under four different activities named the four Es – Education, Engineering, Economics, and Enforcement (Wettermark et al., 2009a) – to provide comparisons with measures used to improve the quality and efficiency of the prescribing of existing drugs across Europe (Godman et al., 2009b,c, 2010, 2011a,b, 2012b,c,f; Wettermark et al., 2009a,b, 2010b; World Health Organisation, 2009; McGinn et al., 2010; Gustafsson et al., 2011; Vončina et al., 2011; Baumgärtel et al., 2012; Bennie et al., 2012; Kalaba et al., 2012; Markovic-Pekovic et al., 2012; Medicine Balance [MEDICIJNBALANS], 2012; van Woerkom et al., 2012); they include:

• **Educational activities** – these range from simple distribution of printed material to intensive strategies including academic detailing and monitoring of prescribing habits usually by professional medical networks. Examples include local, regional, and national formularies, guidance and guidelines including those from Drug and Therapeutic Committees
• **Engineering activities** – organizational or managerial issues to influence change, e.g., quality and efficiency prescribing targets
• **Economic interventions** – financial incentives. These include financial incentives for physicians if they achieve agreed prescribing targets in a class, devolution of drug budgets to local GP groups combined with regular monitoring of prescribing behavior, as well as fines for prescribing costs above agreed limits. Initiatives also include patient co-payments, especially if patients wish a more expensive product than the current reference priced product for the molecule (Anatomical Chemical Therapeutic – ATC – Level 5) or the class/group (ATC Level 3 or 4)
• **Enforcement** – regulations by law such as compulsory International Non-proprietary Name (INN) prescribing, compulsory generic substitution, or prescribing restrictions such as those instigated for patented statins in Austria, Finland, and Norway and the angiotensin receptor blockers (ARBs) in Austria, Croatia, Lithuania, the Republic of Srpska, and Sweden

No attempt has been made to critique the initiatives, including comparing and contrasting the potential influence of the multiple initiatives across the countries and regions to provide future guidance. This is because this would require a thorough analysis of drug utilization patterns alongside associated health policies (Coma et al., 2009; Godman et al., 2009b, 2010, 2011a,b,2012c; Wettermark et al., 2009b, 2010b; McGinn et al., 2010; Garuoliene et al., 2011b; Vončina et al., 2011; Bennie et al., 2012; Kalaba et al., 2012; Markovic-Pekovic et al., 2012; van Woerkom et al., 2012). This will be undertaken in future research projects. However, documented initiatives were used to derive suggested models and potential guidance for all key topics and stakeholder groups to improve the managed entry of new drugs in the future. Initial models and draft guidance were subsequently amended and refined through an iterative process. This involved several rounds with the co-authors until all co-authors were satisfied and agreed with the proposed new model and guidance provided.

## RESULTS

### HEALTH AUTHORITY AND HEALTH INSURANCE COMPANY ACTIVITIES

**Table 1** summarizes some of the health authority/health insurance company activities pre-, peri-, and post-launch up till the end of 2012. Unless stated, the indications are those contained in the EMA marketing authorization (Boehringer Ingelheim, 2011a; Marshall et al., 2013). **Table A1** in the Appendix provides a comprehensive summary of examples from regions and countries across Europe.

**Table 1:** Summary of key activities across Europe to improve the quality and efficiency of prescribing of dabigatran (Godman et al., 2008, 2009a,c, 2011a, 2012d; Holmström et al., 2009; Janusinfo, 2009, 2012a,b; KVH - Aktuell, 2010; Martikainen et al., 2010; Wettermark et al., 2010a,c; Boehringer Ingelheim, 2011b; Gustafsson et al., 2011; Neue Arzneimittel, 2011; Stockholms läns landsting, 2011; Vončina et al., 2011; Keele University School of Pharmacy, 2012; Medicin & Läkemedel, 2012; Midlands Therapeutic Review and Advisory Committee [MTRAC], 2012; Östergötland, 2012; Persson et al., 2012; Davidson et al., 2013).

Timing	Examples of activities among European countries and regions
Pre-launch (principally education)	Swedish counties:
	(A) Östergötland County Council
	• Update of the previous report on the prevalence of atrial fibrillation in Östergötland • Establishing a working party with broad representation from the departments of cardiology and internal medicine, primary health care, representatives from the warfarin polyclinics, epidemiologist and health economists associated with the Drug and Therapeutics Committee (DTC) • Scientific publication of the cost-effectiveness model for dabigatran for the prevention of stroke based on Östergötland by the Centre for Medical Technology Assessment, Linköping University, in collaboration with Östergötland County Council • Consensus action plan agreed 12 months before dabigatran was registered for the prevention of stroke in patients with AF • Recommendation from the DTC to classify dabigatran as a “focus-drug”, i.e., the prescribing unit will be responsible for the cost of the drug. If however patients are entered into the County Council’s quality assessment program, the cost will be borne by the County Council. Decision by the County Council to follow the recommendation of the DTC • Resources for treating patients allocated in the 2011–2012 County Council drug budgets • Communication plan implemented
	(B) Stockholm County Council
	• Systematic and long-term involvement of medical and scientific expertise in the development of guidelines and advise to patients and prescribers through the Regional Drugs and Therapeutic Committee (DTC) and clinical pharmacologists • Extensive pre-launch activities with key messages broadcasted both to the public and to prescribers through websites of the DTC as well as the Swedish Medical Journal • Appreciable number of pre-launch meetings and training sessions with all major physician groups around the key issues and concerns with dabigatran as well as its likely place in care • Production of educational folders regarding dabigatran, slide kits, published articles, and data on the Janus website as well as published information for patients • Forecasting the potential budget impact in 2011 and 2012 ahead of launch and monitoring this in practice • Development of a laboratory method to monitor dabigatran in plasma with LC-MS/MS technology, and recommending sampling in the introductory phase to build a knowledge database. This to be followed by more situation-based sampling to improve patient safety in the future
Peri-launch (principally education)	(A) West Midlands (Region – England)
	• Development of guidance stating that warfarin remains the first-line option for anticoagulation in patients with AF at high risk of a stroke, and primary care trusts (have been replaced by Clinical Commissioning Groups from April 1, 2013) should ensure optimal existing warfarin therapy services – including access to INR clinics, use of computerized decision-support software, and access to drugs for patients who are allergic to warfarin (the latter rare in practice) • In addition in view of the considerable financial implications, dabigatran treatment should only be prescribed for patients: ° with co-morbidities who are adherent to warfarin monitoring and lifestyle requirements but need frequent co-prescribed medications that interact with warfarin and affect the patients’ time in the therapeutic range (TTR) ° who are adherent to monitoring and lifestyle requirements but whose TTR remains unacceptable despite attempts to optimize treatment with warfarin (TTR rates should be set locally)
	(B) Germany
	• Physician Associations stressing when launched that the current knowledge regarding safety with dabigatran was insufficient to answer all questions, and physicians should be careful with prescribing particularly in the elderly • The reporting of deaths from excessive bleeding further endorsed these concerns. As a result, limited prescribing in practice in ambulatory care
	(C) Slovenia
	• Reimbursed in conjunction with a complex price:volume agreement
Post-launch (principally education and enforcement)	(A) Austria (enforcement)
	• Ex ex-ante approval by the head physician of the patient’s social health insurance fund before reimbursement of dabigatran; otherwise 100% co-payment (mirroring other situations) • Renal function has to be assessed and recorded prior to initiation of therapy with dabigatran through determining Creatinine-Clearance (CrCl) levels to exclude patients with severe renal dysfunction (=CrCl < 30 ml/min). In addition during treatment, renal function has to be monitored where a decline is envisaged, e.g., patients with hypovolemia, dehydration, and the use of specific additional medication, and renal function has to be assessed at least once a year in patients aged 75 or older, and/or in patients with compromised renal function
	(B) Finland (enforcement)
	• Reimbursement restricted to patients with risk factors where satisfactory control has not been reached with warfarin; alternatively, warfarin cannot be prescribed due to side-effects or contra-indications • Enforcement at the pharmacy with on average 16 days needed for requests to be centrally reviewed and authorized. Hundred percent co-pay without authorization
	(c) Slovenia
	• Education of all involved specialists and primary care physicians on key safety aspects/adverse events with dabigatran • Prescribing restrictions (Enforcement): • Only reimbursed if initiated by an internist or neurologist and prescribed according to agreed indications, e.g., only reimbursed in patients already on warfarin if they are unstable with TTR < 65 • Patients have to be followed in a tertiary or secondary care anticoagulation center. Patients can be followed in primary care but only if authorized by the tertiary or secondary care center. • Every patient has to be registered in a database and followed by the IT anticoagulation program • Anticoagulation centers have to report once yearly to the tertiary center regarding the number of patients experiencing minor and major bleeding, thromboembolic events, as well as any deaths from bleeding or thromboembolism with dabigatran

Similarly in Australia, the Department of Health and Ageing in the Ministry of Health recently undertook a review of NOACs in the management of stroke risk in patients with AF (Australian Government Department of Health and Ageing, 2012). They recommended to the Pharmaceutical Benefits Advisory Committee (PBAC) the following based on their belief that the net overall benefit of NOACs in clinical practice, and the subsequent impact on cost-effectiveness, is uncertain at this stage:

• Initiating a managed entry scheme taking into account the identified uncertainties while acknowledging the clinical need for effective alternatives to warfarin. This includes the entry price that addresses the uncertainties
• New oral anticoagulants are only reimbursed in patients unable to tolerate warfarin therapy and/or who are unable to obtain satisfactory INR control despite specific measures. This would require a definition of “satisfactory INR control” together with potential price–volume arrangements that address the risk to the Australian Government of use beyond such restrictions

This recommendation has resulted in PBAC undertaking further analysis as it reviews its previous decisions (NPS MEDICINEWISE, 2012).

### PROPOSED MODEL AND ASSOCIATED ACTIVITIES

**FIGURE 1:**
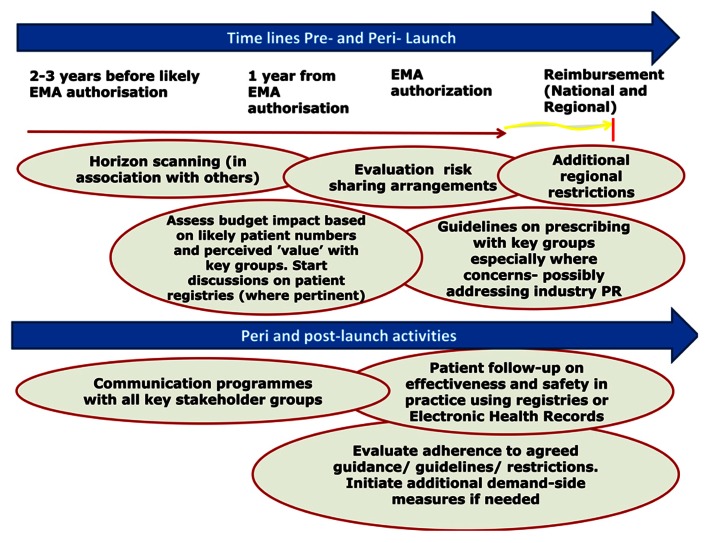
**Proposed model for optimizing the managed entry of new drugs across Europe incorporating national and regional stakeholder groups where pertinent building on the example of dabigatran**.

**Figure 1** outlines the suggested new model to better manage the entry of new drugs in the future. This is based on the extensive knowledge and experience of the co-authors shared across healthcare institutions. This builds on the three pillars of pre, peri-, and post-launch activities (Wettermark et al., 2010a; Godman et al., 2012d).

This starts with horizon scanning activities pre-launch and continues to post-launch monitoring, benchmarking, and registries. Potential activities for each stakeholder group are discussed later in **Table 4**.

There has been a growth in risk sharing arrangements across Europe as health authorities and health insurance companies struggle to fund new premium priced products within available funds (Adamski et al., 2010; Godman, 2011; Jaroslawski and Toumi, 2011; Klemp and Frønsdal, 2011; Cheema et al., 2012; Hirschler, 2012; Jommi, 2012; Siviero et al., 2012; Vogler et al., 2012). Risk sharing has previously been defined as agreements concluded by payers and pharmaceutical companies to diminish the impact on the payer’s budget of new and existing medicines brought about by either the uncertainty of the value of the medicine and/or the need to work within finite budgets (Adamski et al., 2010; Godman, 2011). Consequently, the agreement lies in setting the scope and realizing the mutual obligations amongst both payers and pharmaceutical companies depending on the occurrence of an agreed condition – the “risk,” which varies by situation (Adamski et al., 2010; Godman, 2011).

In view of the concerns with some of these schemes, coupled with the experiences with dabigatran across Europe (**Table 1** and **Table A1** in the Appendix), national and regional health authorities and health insurance companies should consider a number of key issues when appraising risk sharing schemes in the future (**Table 2**). These considerations do not apply to price:volume agreements, straight discounts or rebates, which are easier to administer (Jaroslawski and Toumi, 2011).

**Table 2:** Key issues for health authorities and health insurance companies to consider when appraising risk sharing arrangements proposed by pharmaceutical companies for new drugs.

Key issues regarding risk sharing arrangements
• Validity of the appropriateness of the arrangement(s) for the situation/circumstances in the country/region incorporating current or proposed service delivery arrangements and involving the use of experts • Specificity and transparency of the objectives and scope of the proposed scheme(s) • Novelty of the new drug – including its envisaged health gain, assessment of the effectiveness of current treatments, priority of the disease area, and the translational evidence base • Proportion health authorities will end up funding of a new drug’s development costs through registries post-launch • Data ownership – ideally, all key stakeholders should be involved in the development of any subsequent patient registries. In principal, these should be funded by the manufacturer • Feasibility of IT infrastructure already in place to collect data to monitor the agreement(s) in practice. Alternatively, if new structures are needed, their development costs need to be considered alongside the financial benefits of any proposed risk sharing scheme • Beneficial impact on service delivery and/or safety of the new drug. This should be substantial but has been difficult to prove in Phase III trials • Administrative burden of any proposed risk sharing scheme in relation to the potential overall savings • Likely patient concordance in practice, especially if this has not been fully considered in the proposed scheme(s)

Health authorities and health insurance companies also need to consider a number of key issues before implementing patient registries. Key considerations and issues are shown in **Table 3**. Patient registries can subsequently be used to assess the effectiveness, safety and cost-effectiveness of new treatments in routine clinical care. The nature and extent of data collected will depend on the objectives of any study.

**Table 3:** Key issues for authorities to consider when planning patient registries post-launch.

Events/timing	Key considerations regarding patient registries
Funding and other considerations	*Funding*
	• Explicit and transparent funding arrangements need to be agreed before initiation • Feasibility and potentially pertinence (depending on the nature of the registry) for joint arrangements between authorities and commercial organizations, as seen with the registry for natalizumab in France (Wettermark et al., 2010a; Papeix et al., 2012) and the registries in Italy through AIFA (Italian Reimbursement Agency; Adamski et al., 2010; Wettermark et al., 2010a; Garattini and Casadei, 2011; Jommi, 2012; Siviero et al., 2012) • Any funding arrangements need to be transparent
	*Legal considerations*
	• Compliance with current regulations and legal requirements in each country (although there is a lack of regulations in many countries)
	*Ownership*
	• A priori agreement regarding ownership
	*Endorsement*
	• Registry endorsement by leading research groups/scientific societies, authorities, and patient groups
Timing	• *Timelines*: ensure sufficient time is made available to develop “user friendly” registries that will fully capture all the patient variables of interest and which satisfy the interests of all key stakeholder groups as compromise will be inevitable. This includes: ° Ensuring as far as possible ease of use and acceptability of effort of all those involved ° Ensuring the competence of those entering the data at every data entry point, especially with key issues such as adverse events; enhanced if patients are already experiencing difficulties with their condition such as depression, sleep disorders, fatigue and mobility, as seen in patients with multiple sclerosis. It helps if the disease area is the specialty of those entering the data
	• Data functionality of patient registries need to be considered early pre-launch (**Figure 1**), and time given to recruit personnel competent in computer science and knowledgeable in the major medical issues for the disease area. This will facilitate the development of user friendly screens and data entry to enhance the completeness and accuracy of data entry. In addition, incorporate systems that help detect errors quickly regarding data entry, e.g., trigger tools

Overall, there are a number of activities that each key stakeholder group should consider pre-, peri-, and post-launch to better manage the entry of new drugs. This is especially important where there are potential safety and/or resource issues (**Table 4**). These build on the three pillars and a brief outline of activities discussed in **Figure 1**.

**Table 4:** Key considerations among stakeholder groups to optimize the managed entry of new drugs (Garattini et al., 2008; Godman et al., 2008, 2009c, 2012a,b,d; Wettermark et al., 2008, 2010a,c; Coma et al., 2009; Adamski et al., 2010; Sermet et al., 2010; Gustafsson et al., 2011; Kolasa et al., 2011; Vončina et al., 2011; Cheema et al., 2012; Siviero et al., 2012; Godman and Gustafsson, 2013).

Stakeholder	Key considerations among stakeholder groups to optimize the managed entry of new drugs
Health authorities/health insurance companies/physician associations	*Pre-launch*
	• Plan early for the launch of new drugs especially those that could have an appreciable budget impact and/or safety considerations. This can be through working with countries/regions already engaged in such activities • Work alongside key multi-professional groups including independent pharmacotherapeutic experts such as general practitioners, pharmacists, and clinical pharmacology groups. This will help with critically appraising the potential role and value of new treatments ahead of their launch, as well as with developing robust budget impact models for future forecasts. Where possible, Drug and Therapeutic Committees (DTCs) and expert groups should have a major role to ensure consistent priorities for recommendations across divergent pharmacotherapeutic groups • Work with regulators to: ° Review potential areas of concern with new treatments, especially around safety issues and potential ways to address this ° Check information provided by commercial organizations is comprehensive, addressing any potential publication bias (Melander et al., 2003; Kirsch et al., 2008; Martin, 2012). The need for this should reduce with ongoing activities among pharmaceutical companies to fully disclose trial data (Kmietowicz, 2013) • Plan early for the: ° Incorporation of any pharmacogenetic tests that should to be available when a new “valued” drug is launched to enhance its appropriate use ° Development of any patient registries to assess the effectiveness/safety of new drugs in practice (pharmacovigilance) as well as monitor prescribing against agreed guidance (**Table 3**) ° Any necessary re-designing of services, e.g., anticoagulation services with the launch of new anticoagulants • Regularly assess which products will lose their patent in the coming 1–2 years to help fund new premium priced drugs in the disease area/related disease area – especially with growing resource pressures. These activities will assist financial planning generally • Work with pertinent patient groups especially regarding new treatments that could have serious patient issues to help instigate appropriate educational campaigns for physicians and patients pre- to post-launch. Similarly also with key physicians, including those within DTCs, to develop suitable educational and communication materials including guidelines for physicians
	*Peri-launch*
	• Consider the development of any potential new quality or prescribing indicators together with key stakeholder groups within and across European countries. This includes their assessment in practice, acknowledging that any indicators developed must have content validity, face validity, concurrent validity, construct validity and predictive validity • Include any indicators developed in new guidance/guidelines and, if appropriate, within ongoing financial incentive schemes for physicians to optimize the use of new premium priced drugs at launch • Be critical of any proposed risk sharing arrangements using the criteria summarized in **Table 2** – mindful that such arrangements post-launch could facilitate reimbursement and funding of new premium priced drugs (**Table A1** in the Appendix – e.g., Netherlands and Slovenia) • Continually check likely launch dates for new treatments with pertinent pharmaceutical companies to improve financial planning
	*Post-launch*
	• Use administrative and/or medical databases to compare “real world” patients with those included in Phase III RCTs in terms of their clinical features, treatments, and potential outcomes to further refine prescribing guidance and/or reimbursed prices especially if greater co-morbidity in “real world” patients (Joppi et al., 2013) • Build in regular reviews of any reimbursement/funding/guidance especially as more data becomes available, e.g., more recent data challenging “no patient monitoring” with dabigatran especially if “no patient monitoring” was built into submitted economic analyses • Monitor physician adherence to any agreed guidance/reimbursement restrictions and potentially instigate academic detailing and other activities where continued concerns with prescribing
Physicians	*Peri-launch*
	• Work with health authorities and health insurance companies pre-launch to critically review new treatments, especially where there are concerns with patient safety, to help enhance their appropriate use at launch and their retention on the market • Provide guidance to health authorities and health insurance companies regarding optimal patient populations that maximize the value of new drugs, as well as potential quality/prescribing indicators • Provide input into discussions on the potential value of pertinent pharmacogenetic tests that may help optimize the use of new drugs post-launch • Help with the development of educational materials for physicians and patients peri- and post-launch including the development of any clinical guidelines based on agreed guidance • Assist with the design of any patient registries prior to launch, and follow this up after launch (**Table 3**). This can also include programs that measure drug sequencing against any agreed guidance • Help authorities critically assess proposed risk sharing arrangements, especially regarding the administrative burden and other key issues (**Table 2**) • Assist hospital and ambulatory care DTCs with critically evaluating new treatments, as well as with the planning of any interface arrangements to improve the co-ordination of care between primary and secondary care physicians post launch
	*Post-launch*
	• Provide input into any patient registries (**Table 3**) to help assess the true value of the new drug especially where there are concerns with safety in a wider co-morbid population post launch than those enrolled into Phase II and III trials • Provide input when clinical guidelines are revised as more data becomes available
Patient organizations	*Pre-launch*
	• Provide input to health authorities and health insurance companies pre-launch regarding any safety and effectiveness issues for new drugs from the patients’ perspectives • This includes any pertinent pharmacogenetic tests that help optimize the use of new drugs to patient populations where the benefit:risk ratio (and hence “value”) is maximized
	*Pre- and peri-launch*
	• Provide input into the design and distribution of any patient information regarding new drugs, especially where potential safety issues, pre- and peri-launch • Provide input into the design of any quality/prescribing indicators for new drugs especially where there are issues of safety and sequencing as well as where compliance is likely to be a concern
	*Post-launch*
	• Help further refine information for patients as more knowledge becomes available about the new drug, especially regarding key side-effects and their implications • Help disseminate factual information to patients if pertinent, especially where there are exaggerated claims unduly raising expectations among patients or where issues of side-effects have not been adequately disseminated
Commercial organizations	*Pre-launch*
	• Interact early with pertinent health authorities and health insurance companies, especially for new premium priced drugs, to review key comparator and outcome data to include in Phase II/III clinical trials. Comparator and outcome data will depend on the disease area and target prices. Included in this should be discussions regarding resource issues and budget impact at launch to aid planning, acknowledging the particular characteristics of each market • This may include discussions on study design with increasing knowledge of pharmacogenomics and the implications for subsequent trial designs with potentially smaller populations (this will be explored further in future papers) • Provide health authorities and health insurance companies with all relevant data in a timely fashion pre-launch, rather than selective data, to aid decision making and reduce scepticism. This is important to address current concerns that manufacturers are still hiding/not providing data that potentially reduces the value of their product (Martin, 2012); although some companies are now addressing this (Kmietowicz, 2013) • Relevant data includes key adverse event data or pharmacokinetic data - especially if there are concerns about potential claims in practice as seen with concerns with the “no requirement for patient monitoring” with dabigatran • Be pragmatic when planning target prices taking into account key decision making criteria for the pertinent country or region, including either cost/QALY considerations or clinical data requirements for new drugs to be seen as innovative or adding clinical value. This includes any discounts or rebates as part of any risk sharing arrangements (**Table 2**), acknowledging that the majority of new drugs are seen as similar by payers, with only a minority seen after critical evaluation to have added patient benefits compared with existing standards. These considerations have grown in importance with ongoing resource pressures, i.e., mindful of opportunity cost considerations within health authorities and health insurance companies
	*Peri- and post-launch*
	• Resist the urge to over promote new drugs especially to the public where there are safety issues, thereby reducing the potential for further restrictions/early withdrawal • Potentially monitor and refine risk sharing arrangements as more data becomes available

### EMA AND FDA ACTIVITIES

The low mean bioavailability of dabigatran (Douxfils et al., 2012; Mannuci et al., 2012; Ten Cate, 2012), as well as studies demonstrating considerable variation in plasma drug concentrations in practice, led the FDA in 2010 to explore the relationship between dabigatran concentrations in plasma and the risks of suffering a stroke or major bleeding (Thompson, 2010; Ten Cate, 2012). These publications also demonstrated it is important to avoid too low or too high levels of dabigatran (Mismetti and Laporte, 2010; Thompson, 2010). Consequently similar to warfarin, certain patients on dabigatran and other NOACs should be monitored to reduce potential side-effects (Mismetti and Laporte, 2010; Douxfils et al., 2012; Mannuci et al., 2012; Ten Cate, 2012).

The EMA in their *Risk Minimization Plan* for dabigatran issued in 2011 also defined a cut-off for the risk of bleeding with the 150 mg *bid* regimen of 200 ng/mL dabigatran in plasma at C_trough_ (Heidbüchel et al., 2011).

## DISCUSSION

Dabigatran and the other NOACs are the result of a long search for an alternative to warfarin to prevent strokes in patients with AF. However, the weighing of the advantages and disadvantages associated with dabigatran, especially in the elderly with poor renal function, needs to be judged carefully and handled appropriately alongside the additional acquisition costs of dabigatran. These challenges led to an extensive range of activities among national and regional health authorities, health insurance companies, and physician associations across Europe pre-, peri-, and post-launch to enhance its appropriate use (**Table 1** and **Table A1** in the Appendix).

The main medical concerns were the risk of excessive bleeding in elderly patients with AF with no known antidote, variable plasma drug concentrations in practice exacerbated by low bioavailability, and the dependence on renal elimination of the active metabolite (Baetz and Spinler, 2008; Malmström, 2009; Legrand et al., 2011; Liesenfeld et al., 2011; Banerjee et al., 2012; Douxfils et al., 2012; Godman et al., 2012d; Harper et al., 2012; Huisman et al., 2012; Mannuci et al., 2012; Ten Cate, 2012; Marshall et al., 2013). Cases of major bleeding and deaths were seen with dabigatran soon after its launch (Malmström, 2009; Institute for Safe Medication Practices, 2011; EMA, 2011; Legrand et al., 2011; Wood, 2011; Garber et al., 2012; Godman et al., 2012d; Harper et al., 2012; Mannuci et al., 2012; Lothian Prescribing Bulletin, 2012; Marshall et al., 2013). The EMA reported on November 6, 2011 that there had already been 256 spontaneous reports of serious bleeding resulting in deaths in the EudraVigilance database (EMA, 2011).

**Table 1** and **Table A1** in the Appendix document the extensive range of activities initiated across Europe. These include educational activities pre-launch in Stockholm County Council, Sweden, as well as post-launch activities among regions and localities in Germany, Spain, Sweden, and the UK. There were also prescribing restrictions in some countries alongside the development of shared care protocols between ambulatory and hospital care to improve interface management and enhance the subsequent quality of care (Godman et al., 2012e). It is suggested that these activities reduced subsequent bleeding among patients in practice, especially among those with poor renal function and, as a result, potentially helped preserve the availability of dabigatran across Europe. This is unlike that situation seen with a number of drugs described earlier including zimelidine, COX-II inhibitor drugs, cerivastatin, and rimonabant (Fagius et al., 1985; Carlson, 1999, 2000; Friedman et al., 1999; FDA, 2002; Merck, 2002, 2004; MHRA UK, 2004; Calfee and Pinell, 2005; European Medicines Agency [EMA], 2009; Moreira et al., 2009; O’Shaughnessy, 2009; Eichler et al., 2011; Ioannides-Demos et al., 2011; Kappos et al., 2011; Keegan, 2011; Dietrich and Horvath, 2012). However, it is difficult to substantiate this without definite research. Having said this, reimbursement of dabigatran has recently been rejected in Poland due to concerns with excessive bleeding and deaths (**Table A1** in the Appendix).

There have also been issues with the additional costs of dabigatran versus warfarin at GB£919.80 (€1060) per patient (UK) given the growing prevalence of AF with currently over 4.5–6 million patients across Europe and rising (Lloyd-Jones et al., 2004; Stewart et al., 2004; Camm et al., 2010; Pink et al., 2011; Mannuci et al., 2012; Marshall et al., 2013). However, there is less of a budget differential in Sweden (Davidson et al., 2013). These combined issues led to (i) prescribing restrictions in some countries alongside prior authorization schemes, e.g., Austria, Belgium, Finland, NHS Bury (initially), Slovakia, and Slovenia, (ii) delays with reimbursement in others including Croatia (still undergoing review), the Netherlands, Norway (only just reimbursed), and Portugal (150 mg); as well as (iii) price:volume and other agreements (risk sharing) to lower the price of dabigatran, e.g., Ireland, the Netherlands, and Slovenia as well as potentially in Croatia (**Table 1** and **Table A1** in the Appendix). These concerns have also resulted in dabigatran not being reimbursed/not preferred as an alternative in some countries and regions including Estonia, Lithuania, Lothian Health Board (Scotland), NHS Cornwall Community Health, the Republic of Serbia, and Turkey (**Table A1** in the Appendix). Prescribing restrictions and risk sharing arrangements are no doubt preferred by manufacturers versus not having their drugs reimbursed.

The weighing up of the benefits and concerns with dabigatran make it increasingly important for European countries and regions to develop and refine models to further improve the managed entry of new premium priced drugs, even if they do not have a tradition of Health Technology Assessment (HTA). The alternative could be reduced resources to fund new drugs in the future, especially with a growing elderly population, which is already happening (Garuoliene et al., 2011a,b; Godman et al., 2011c, 2012b; Taylor, 2011). As mentioned previously, budgetary pressures are growing as a result of the number of new biological drugs in development (EvaluatePharma, 2012; Godman, 2013) including new cancer drugs (Nagle et al., 2008; National Cancer Institute, 2010; Sullivan et al., 2011; Mullard, 2012), which are now costing up to US$10,000–25,000 (€7580–18,960) per patient per month (Selyukh, 2011; Yukhananov, 2011; Kaiser, 2012; UK Medicines Information, 2012; UKMi Medicines Information, 2013). Such models may also reduce the possibility of new drugs such as dabigatran being withdrawn from the market due to a greater level of side-effects in a wider co-morbid population than that included in the clinical trials (Joppi et al., 2013). None of these alternative scenarios are in the best interests of any key stakeholder group.

Moreover, it is critical that health authorities and health insurance companies take full advantage of the increasing availability of standard drugs as generics to help fund increased volumes and new premium priced drugs in the future (Frank, 2007; Jack, 2008; Godman et al., 2012a,b,f). For example, expenditure on proton pump inhibitors (PPIs) and statins would have been GB£449 million (€520 million) higher in Scotland in 2010 without appreciable demand-side measures enhancing the prescribing of low cost generics for its 5.2 million population (Bennie et al., 2012; Godman et al., 2012b). This activity is driven by global sales of products likely to lose their patents between 2008 and 2013 estimated at US$50–100bn (€38–76bn), and considerably higher for subsequent years (Frank, 2007; Jack, 2008; Godman et al., 2012b,f), out of global sales of pharmaceuticals estimated at US$820bn (€625bn) in 2009 (EATG, 2009).

The next stage of our research will be to assess the influence of the plethora of health authority and health insurance company activities (**Table 1** and **Table A1** in the Appendix) on subsequent utilization of dabigatran and other NOACs, alongside ongoing reforms, to further refine the suggested model (**Figure 1**). This should also help with future recommendations regarding potential demand-side measures that could be introduced to further improve the managed entry of new drugs, based around the four Es (Wettermark et al., 2009a). This includes the implications for all key stakeholder groups (**Table 4**). We are already seeing health authorities and health insurance companies monitor the effectiveness and safety of patients prescribed dabigatran and other NOACs, and this will grow.

In the meantime, we hope we have demonstrated why it is imperative that health authorities and health insurance companies continue to develop and refine new models to better manage the entry of new drugs in the future. In addition, we hope we have provided direction to all key stakeholder groups based on our considerable experience to further stimulate this debate in this critically important area. This especially as the constant introduction of new premium priced drugs is seen as the greatest challenge to the continued provision of equitable and comprehensive healthcare in Europe (Garattini et al., 2008; Godman et al., 2012d).

## CONCLUSION

There have been multiple activities pre- to post-launch among authorities across Europe to improve the prescribing of dabigatran, especially in elderly patients where there are concerns with their renal function. In addition, address potential concerns with the budget impact of dabigatran through for instance price:volume agreements and prescribing restrictions.

We believe and recommend, based on the experiences with dabigatran and other new premium priced drugs, that it is essential for authorities to develop new models to better manage the entry of new drugs in the future (**Figure 1**). This is becoming critical given the number of new premium priced drugs in development.

Critical activities for health authorities and health insurance agencies pre-launch in the future involve horizon scanning and budget planning activities. This includes identifying products likely to lose their patent within the next 1–2 years. In addition, educational materials and clinical guidance need to be developed pre-launch with the help of physicians and patient groups. Key peri-launch activities include developing prescribing indicators for new treatments as well as the critical appraisal of any proposed risk sharing arrangements, assessed against the criteria documented in **Table 2**. Essential post-launch activities include monitoring of prescribing against agreed guidance. Increasingly also entering patients into registries to monitor the effectiveness and safety of new drugs in wider patient populations having considered key issues (**Table 3**).

Without such models, authorities may well struggle to maintain the European ideals of equitable and comprehensive healthcare as well as ensuring funding for new “valued” treatments in target populations. Consequently, the development of new models to better manage the entry of new drugs should be in the interest of all key stakeholder groups.

## Conflict of Interest Statement

There are no conflicts of interest from any author. However, the majority of authors are employed by health authorities, health insurance companies, universities, or Physician Associations or are advisers to them. The content of the paper and the conclusions are those of each author and may not necessarily reflect those of the organization that employs them.

## Appendix

**Table A1:** Examples of health authority and health insurance company activities regarding dabigatran for the prevention of stroke in adults with non-valvular AF among European countries to the beginning of 2013 (building on EU marketing authorisation – (Boehringer Ingelheim, 2011a; Marshall et al., 2013).

Country	Date dabigatran reimbursed for AF	Summary of activities
Austria (Godman et al., 2008, 2009a; Wettermark et al., 2009a; Vončina et al., 2011; Therapie Tipps, 2012)	February 2012	*Post-launch*
		Enforcement – Ex ex-ante approval by the head physician of the patient’s social health insurance fund before reimbursement of dabigatran; otherwise 100% co-payment (mirroring other situations). This is now fully automated, with the first prescription typically taking approximately 30 min to approve
		The renal function has to be assessed and recorded prior to initiation of therapy with dabigatran through determining Creatinine-Clearance (CrCl) levels to exclude patients with severe renal dysfunction (= CrCl < 30ml/min). In addition during treatment, renal function has to be monitored where a decline is envisaged, e.g., patients with hypovolaemia, dehydration and the use of specific additional medication, and renal function has to be assessed at least once a year in patients aged 75 or older, and/or in patients with compromised renal function. Otherwise 100% co-payment
		Health Insurers (WGKK – Vienna) have also stated that patients who are well adjusted on Vitamin K antagonists should not be switched to dabigatran as there is no additional clinical benefit, enhanced by currently no known antidote.
Belgium (Federal Agency for Medicines and Health Products (FAMHP), 2011)	August 2012	(i) Reimbursement
		• 2 × 150 mg per day based on the SPC and the patient is not subject to one of the following: ° Older than 80 years; ° Treated with verapamil; ° Serious renal insufficiency • 2 × 110 mg dose – reimbursed in the absence of serious renal insufficiency but without age or verapamil restrictions
		(ii) Education (pre-launch)
		• 25th November 2011, the Federal Agency for Medicines and Health Products issued an update concerning the risk of fatal bleeding with dabigatran. This was based on the CHMP’s recommendations at the EMA that precautionary measures need to be strengthened in the case of renal insufficiency. Physicians were also informed of the CHMP recommendations in a letter • In March 2012, the Belgian Centre for Pharmacotherapeutic Information stated that: ° Based on the currently available studies, dabigatran and rivaroxaban appear to be as efficacious as warfarin in the prevention of thrombo-embolism in non-valvular atrial fibrillation ° Their risk-benefit ratio did not seem to be superior to VKAs when VKAs were used in appropriate doses within the INR range ° Awaiting additional studies, and taking into account the limited data and high price of NOACs, VKAs remain the first choice for many patients ° Dabigatran and rivaroxaban can be alternatives in patients for whom treatment with a VKA is difficult to control ° In the absence of comparative studies between dabigatran and rivaroxaban, there are no arguments to prefer one product to the other ° NOACs can lead to specific medicine interactions (but to a lesser extent than with VKAs) and to over-dosing when renal function declines (need to pay attention in the elderly).
		Peri-launch (Enforcement)
		• Dabigatran was reimbursed as a chapter IV medicine in AF patients. A chapter IV medicine can only be prescribed subject to prior approval from the advising physician of the patient’s health insurance fund - otherwise a 100% patient co-payment applies • Reimbursement is restricted to one pack of 60 × 150 mg and three packs of 180 × 150 mg, with a maximum validity period of 300 days • Reimbursement can subsequently be extended for renewable periods of 360 days for four packs of 180 × 150 mg per period • Concomitant reimbursement of dabigatran with another oral anticoagulant is not allowed
		Post-launch (Education, Engineering)
		• As part of the risk management plan for dabigatran, a warning card/patient card was made available for patients to keep with them at all times • By giving this card to patients, it is envisaged that physicians and pharmacists will enhance the appropriate use of dabigatran and limit potential side effects - especially as the patient is encouraged to show this card to every physician, pharmacist or other health professional
Croatia	Under evaluation	Reimbursed for the prevention of venous thromboembolism in patients undergoing hip or knee surgery, and only in hospitals, with prescriptions traced in hospitals if abuse is suspected.
		Under consideration for the prevention of stroke and systemic embolism in adult non-valvular AF patients including price:volume agreements and/ or co-payments. There are also ongoing discussions regarding safety issues
England (Horsley, 2010; Elton et al., 2011; Keele University School of Pharmacy, 2012; Midlands Therapeutic Review and Advisory Committee [MTRAC], 2012; National Institute for Health and Clinical Excellence, 2012; Marshall et al., 2013; NHS Improvement. Guidance on Risk Assessment and Stroke Prevention for Atrial Fibrillation (GRASP – AF), 2013) Coventry and Warwickshire Area Prescribing Committee (2012); Interface – A monthly medicines and prescribing bulletin for healthcare professionals in East Lancashire focusing on new therapies (2011); East Lancashire NHS Medicines Management Health Economy New Medicines and Treatments Group - Dabigatran for prevention of stroke in non-valvular atrial fibrillation (2012); NHS Cumbria, NHS Lancashire (2012a),b;	August 2011	*Post-launch*
		(A) National – NICE:
		Dabigatran is recommended in line with the licenced indication, with the decision whether to start treatment made after an informed discussion between clinicians and patients about the risks and benefits of dabigatran versus warfarin.
		For patients already on warfarin, the potential risks and benefits of switching to dabigatran should be considered in light of current INR control.
		(B) Regions (Midlands – MTRAC) – Education
		Guidance stating that warfarin remains the first-line option for anticoagulation in patients with AF at high risk of a stroke, and PCTs should ensure optimal existing warfarin therapy services – including access to INR clinics, use of computerised decision-support software, and access to drugs for patients who are allergic to warfarin (the latter is rare in practice)
		In view of the considerable financial implications, dabigatran treatment should only be prescribed for those patients:
		• with co-morbidities who are adherent to warfarin monitoring and lifestyle requirements but need frequent co-prescribed medications that interact with warfarin and affect the patients’ time in the therapeutic range (TTR) • who are adherent to monitoring and lifestyle requirements but whose TTR remains unacceptable despite attempts to optimise treatment with warfarin (TTR rates should be set locally)
		Alongside this, patient follow-up via agreed shared care protocols with ongoing monitoring of prescribing costs and feedback from Pharmaceutical Advisers.
		(C) Localities
		(i) Coventry and Warwickshire Area Prescribing Committee (Education, engineering):
		• Dabigatran should only be initiated by a specialist • Follow on prescribers should receive a checklist from the initiating specialist indicating patients are suitable for dabigatran and have received appropriate guidance from the specialist • No follow-on prescribing if checklist is unavailable from the specialist
		(ii) East Lancashire (Education):
		Initially not approved (October 2011); but subsequently approved for usage in January 2012. As part of this:
		• Patients currently stable on warfarin therapy should not be considered for dabigatran. • Dabigatran should only be considered for prescribing by appropriate specialists (including GPs initiating therapy as part of specially commissioned anti-coagulation services) • Dabigatran should only be considered as an alternative to warfarin for stroke prevention in AF patients in the following: 1. Patients for whom warfarin is contraindicated or not tolerated or not suitable (e.g., Mental/cognitive impairment) – NOTE: If warfarin is contra-indicated due to increased bleeding risk then dabigatran would also be contra-indicated. 2. Patients who are poorly controlled on warfarin, i.e. a clinical judgement based on patient reviews relating to the extent INR results are outside of the target therapeutic range (TTR). If dabigatran is considered as a suitable alternative, prescribers must fully document the rationale
		(iii) NHS Bury
		• Greater Manchester Medicines Group [GMMMG] and the Greater Manchester and Cheshire Cardiac and Stroke Network [GMCCSN] agreed joint guidance for the use of dabigatran behind warfarin. This resulted in NHS Bury establishing a “Gateway” whereby GPs had to seek permission from NHS Bury to prescribe dabigatran • This resulted in very few requests, with even fewer requests granted due to ongoing clinical and economic concerns. • Following NICE advice [TA249], NHS Bury have instigated a number of educational activities with Consultants and GPs including joint symposia and the production of local prescribing guidance • In addition, encouraging GP practices to work with the NHS Improvement Tool – GRASP – AF (Guidance on Risk Assessment and Stroke Prevention for Atrial Fibrillation) to identify patients with AF at high risk of a stroke that are currently not properly anti-coagulated as potential candidates for NOACs
		(iv) NHS Cornwall Health Community (Education)
		• Prescribing of new NOACs discussed at the Area Prescribing Committee • Development of prescribing guidance stating that warfarin remains first line option but noting that dabigatran can be considered for patients with AF not taking warfarin for reasons of intolerance, previous significant adverse effects with warfarin, interactions, circumstances where routine monitoring may be impractical, and those with AF with poor INR control on warfarin • Prescribing of dabigatran (and other new NOACs) monitored monthly and reported back to GPs during forum meetings between prescribing advisers and GPs
		(v) NHS Lancashire/ NHS Cumbria (Education, economics):
		• Developed a consensus statement for NOACs together with NHS Cardiac and Stroke Networks in Lancashire and Cumbria. This included: ° On the balance of risks and benefits suggest warfarin is considered for high risk atrial fibrillation patients i.e., those with CHADS2 > 2. Where CHADS2 < 2, risk assess using CHA2DS2VASc and reconsider the need for anticoagulation. ° NOACs are recommended as an option where warfarin is either contraindicated or where the patient has a documented hypersensitivity to or intolerance of coumarin anticoagulants severe enough to cause treatment withdrawal. In situations where repeated INR testing/monitoring may be impractical, the use of a NOAC should be considered. However, increased bleeding risk as a contraindication to warfarin also applies to NOACs. ° An assessment of bleeding risk should be considered before starting anticoagulation. The HAS-BLED score is recommended to assess bleeding risk in AF patients, however, remember stroke and bleeding share risk factors. ° For patients who are already taking warfarin, the potential risks and benefits of switching to a NOAC should be considered in light of their level of INR control. Where patients are spending over 65% of their time in the therapeutic range (TTR) on warfarin, there is likely to be little benefit in switching to a NOAC. ° For new patients requiring anticoagulation, warfarin should be considered and, after an informed discussion with the patient, should be initiated with its effectiveness assessed after 3 months of treatment. ° The decision about whether to start a NOAC should be made after an informed discussion between the clinician and the patient about the risks and benefits of NOACs compared to warfarin. Patient Decision Aids are being developed by the Medicines and Prescribing Centre (NPC of NICE) and their use is endorsed by this consensus group. ° Whilst NOACs have a shorter half-life than warfarin, they have no simple antidote. Whilst they don’t need anticoagulation monitoring, there is no standardised easy way of measuring their effectiveness. Conversely, warfarin has been in use for over 60 years, its effects are measurable and can be rapidly reversed in the event of major bleeding • NHS Lancashire and NHS Cumbria also produced guidance for the prescribing of dabigatran. This included: ° Contraindications ° Advice on how to convert patients from warfarin to dabigatran and conversion from dabigatran to warfarin ° Interactions with other medicines ° Advice on the discontinuation of dabigatran based on renal function • Potential rebate schemes were initially discussed to reduce the costs of dabigatran to NHS Lancashire. However, this was not taken forward with the change to a Clinical Commissioning Group
Estonia	Not reimbursed	Dabigatran rejected for this indication as not seen as sufficiently cost-effective versus warfarin in view of its high acquisition costs
Finland (Martikainen et al., 2010; Godman et al., 2011a)	April 2012	*Post-launch (enforcement)*
		Reimbursement restrictions (Enforcement) – limiting the reimbursement of dabigatran to patients with risk factors where satisfactory control has not been reached with warfarin; alternatively, warfarin cannot be prescribed due to side-effects or contra-indications.
		Enforcement at the pharmacy with on average 16 days needed for requests to be centrally reviewed and authorised. 100% co-pay without authorisation.
France (Sermet et al., 2010; Godman et al., 2012a; Haute Authorite de Sante – Commission de la Transparence, 2012)	ASMR Rating February 2012	*Peri-launch*
		Dabigatran classified as ASMR V (no additional therapeutic value) compared with current therapies for the prevention of strokes in adults at risk who have “non-valvular atrial fibrillation” and are considered to be at risk of stroke.
		*Post-launch*
		(a) April 2012 – Education:
		(i) Publication of information about dabigatran from the authorities including a warning from the medicine agency ANSM (ex afssaps):
		• risk of haemorragia and overdose • absence of biological tests and an antidote
		(ii) Publication of advice for (1) change of prescribing from or to other anticoagulants, (2) patients undergoing surgery
		(b) May 2102
		Translation of the latest advice from the EMA
		Once reimbursed, patients will be followed up to assess the effectiveness and safety of dabigatran in practice (pharmacovigilance)
Germany (IQWiG, 2010; *Kreatinin-Clearance* Rechner (Creatinine Clearance calculator), 2013)	August 2011	*Peri- and post-launch*
		Activities (education and engineering) included the following:
		• Information from State and National Physician Associations to ambulatory care physicians stressing concerns and potential sanctions with “off label” use in AF prior to its licensing approval • Physician Associations stressing when launched that the current knowledge regarding safety with dabigatran was insufficient to answer all questions, and physicians should be careful with prescribing particularly in the elderly • The reporting of deaths from excessive bleeding further endorsed these concerns. As a result, limited prescribing in practice in ambulatory care • A warning letter from Boehringer Ingelheim following issues and deaths from excessive bleeding in Japan. In the letter, BI stated that patients should not be prescribed dabigatran if their creatinine clearance is <30 ml/min and/ or significant renal impairment. In addition, the need to monitor renal function when using dabigatran especially in patients prone to poor renal function or where renal function is deteriorating (measured using the Cockroft-Gault formula) • Information to patients about anti-coagulation in general including dabigatran
Ireland (National Centre for Pharmacoeconomics, 2011; Burke, 2012; Health Service Executive (HSE) Ireland. Primary Care Reimbursement Service (PCRS) Online Services, 2012)	July 2012	*Peri- and post-launch*
		August 2011: The National Centre for Pharmacoeconomics (NCPE) stated that “dabigatran etexilate could be considered a cost effective treatment for the prevention of stroke and systemic embolism for adult patients with atrial fibrillation and one or more of the specified risk factors. However there are uncertainties associated with some of the clinical input data and the model assumptions in addition to the considerable opportunity cost, in the region of €13 million over 10 years.” In view of this a reduction in price is recommended to ensure value for money for the health service in Ireland (HSE).
		November 2011: A HSE Statement advises that the drug will not be reimbursed if prescribed for any *new* patients for SPAF (Stroke prevention in patients with AF).
		July 2012: A HSE Statement states that:
		Warfarin is the recommended first line agent for stroke prevention in atrial fibrillation. Dabigatran should be reserved for:
		• Existing patients on warfarin with poor INR control despite adhering to monitoring and lifestyle requirements. Documentation of attempts to optimise warfarin therapy is required. • Existing patients who require regular periodic treatment with medicines that are known to interact with warfarin • Patients with a documented allergy to warfarin
		As part of the implementation, the physician responsible has to make a specific application for each patient to HSE. Otherwise, pharmacists will not be reimbursed for dispensing dabigatran to patients for SPAF without prior reimbursement approval (enforcement)
		October 2012: Following the NCPE pharmacoeconomic assessment (August 2011), the manufacturer reduced the price of dabigatran. At this revised price, the NCPE now considers dabigatran to be cost effective in this situation
Italy (Adamski et al., 2010; Cheema et al., 2012; Jommi, 2012; Siviero et al., 2012)	Undergoing evaluation	*Pre-launch*
		• Educational meetings at regional and national level with different stakeholders to (i) identify possible prescriber(s) (cardiologists only or GPs as well) and the target population; (ii) define a sustainable price and (iii) define the features of a follow-up programme for patients treated with dabigatran • Forecasting the potential budget impact in the first and second year post-launch with the help of key stakeholder groups
		*Post-launch*
		• Planning a national registry containing details of the clinical characteristics, current pharmacological treatments, and potentially outcomes of patients with AF prescribed dabigatran • To limit the prescribing to specialists only (cardiologists, others) and to involve the GPs in the follow-up of treated patients; • To network prescribers (specialists) and GPs as well as instigate educational activities among specialists (prescribers) and GPs according to their needs
Lithuania	Not reimbursed	Reimbursement was rejected at the end of 2012 as dabigatran was not seen as sufficiently cost-effective versus warfarin
Netherlands (Medicine Balance [MEDICIJNBALANS], 2012)	December 2012	*Peri- to post-launch(education, economics)*
		• The National Health Council advised the Ministry of Health to reimburse dabigatran (and rivaroxaban) but additional research concerning the specific Dutch situation was needed. The research should be performed in the real world population seen in clinical in practice • The Ministry of Health subsequently asked prescriber organizations to establish a guideline for the safe and responsible introduction of dabigatran. The guideline to contain a protocol for calamities, prioritized patient groups (which groups are high priority) and instructions to contribute to a patient registry • The national guideline has now been produced. Each hospital is expected to produce its own protocol based on the national guidance as well as make arrangements with GPs and ambulance services • As part of reimbursement, the Ministry of Health sought a price-volume agreement with the manufacturer • In addition, a review has been made available by the Institute for the Rational Use of Medicines giving an overview of the current evidence surrounding dabigatran in AF for physicians. Experts are being used to initiate online discussions on the new website from this institution to further enhance appropriate prescribing (Education)
Norway	January 2013	*Peri-launch*
		• Dabigatran was recently assessed by the National Medicines Agency of Norway (NOMA) and considered to be cost-effective for the prevention of stroke in adults with non-valvular AF • The Ministry (as of October 2012) favoured the reimbursement of dabigatran from 1 January 2013, and the Budget Bill was voted in on 5 December 2012 • No ongoing activities such as price: volume agreements or educational activities
Poland (Agencja Oceny Technologii Medycznych Rada Przejrzystości, 2012)	Not reimbursed	The Transparency Council of the Polish HTA Agency assessed dabigatran for its potential inclusion in the national reimbursement list
		The Transparency Council subsequently rejected reimbursement due principally to safety concerns. The Council was concerned about the number of serious bleeds and deaths that had already occurred in the United States and New Zealand soon after its launch in these countries.
Portugal	August 2011 for 110 mg (when PL granted)	• Reimbursed for 110 mg for the prevention of stroke in patients with atrial fibrillation once approved by EMA, as already reimbursed for prophylaxis in patients undergoing hip and knee surgery (current legislation in Portugal). As a result, appreciably increasing utilisation of 110 mg strength • 150 mg is currently not reimbursed for AF, but is undergoing evaluation alongside an accompanying pharmacoeconomic study versus warfarin which was submitted by the manufacturer to demonstrate the cost-effectiveness of 150 mg dabigatran versus warfarin • Ongoing activities to lower the reimbursed acquisition cost (price) for AF patients for the 110 mg strength
The Republic of Serbia	Not reimbursed	Dabigatran is currently not reimbursed in Serbia principally due to concerns with its price/ budget impact versus warfarin for the prevention of stroke in patients with AF and the perceived limited benefits in practice.
Republic of Srpska	Not currently reimbursed	• The manufacturers made a submission to the Health Insurance Fund (HIF) • The submission has been reviewed by the HIF and proposed for inclusion in the reimbursement list • However to date, there has been no decision to include dabigatran in the reimbursed list of drugs
Scotland (NHS Tayside, 2011; Fife Area Drug and Therapeutics Committee (2012); Health Improvement Scotland (2012); NHS Highland - The Pink One (2012); Scottish Medicines Consortium, 2011; Oral anticoagulants, 2012; Marshall et al., 2013)	August 2011	*Peri-launch*
		National (SMC) – Dabigatran is accepted for use in accordance with the approved indication as it was seen to be at least as effective as standard oral anticoagulation at preventing stroke or systemic embolism and was not associated with an increased risk of major bleeding.
		*Post-launch (among the Health Boards) (educational)*
		(a) Fife (December 2011)
		Dabigatran should only be prescribed in line with advice from Healthcare Improvement Scotland, i.e., on balance of risks and benefits of dabigatran, warfarin remains the anticoagulant of clinical choice for moderate or high risk atrial fibrillation patients (CHA2DS2-VASc = 2) with good INR control, and clinicians should only consider prescribing dabigatran in patients with:
		• poor INR control despite evidence that they are complying, or • allergy to or intolerable side effects from coumarin anticoagulants
		(b) Highlands (December 2011)
		• Warfarin remains the anticoagulant of choice as a greater rate of GI bleeding and GI symptoms with dabigatran • In addition, much easier to manage major bleeding in patients with warfarin as no licensed product available to reverse bleeding with dabigatran (unlike warfarin) • If needed, dabigatran should only be started when patient’s INR has dropped below 2
		(c) Tayside (December 2011)
		• Prescribing should be restricted to patients with poor INR control on warfarin, or with allergy to or intolerable side-effects from coumarin anticoagulants • Under the guidance, anticoagulant clinics in NHS Tayside will identify eligible patients and make contact with relevant GPs with the decision to transfer patients resting with GPs • In addition, prescribers should note recent MHRA advice that renal function should be assessed in all patients before starting dabigatran. While on treatment, renal function should be assessed at least once a year in patients >75 years and when a decline in renal function is suspected
		(d) Lothian (May 2012 Bulletin)
		• Dabigatran classified as “not preferred as suitable alternatives exist.” • The main concerns were safety and the management of any bleeding episodes when they occur
Slovakia	April 2012	*Peri-launch*
		• Dabigatran was assessed by the Categorisation Committee at the Slovak Ministry of Health and considered cost-effective for the prevention of stroke in adults with non-valvular AF. The incremental cost-effectiveness ratio (ICER) of dabigatran versus standard treatment was estimated at €17,437, which is below the Slovakian acceptable threshold (€18,000 per QALY gained). The sensitivity analysis consistently demonstrated the cost-effectiveness of dabigatran. • Only reimbursed if prescribed by cardiologists, neurologists or internists in line with the approved indications (enforcement). One of the following requirements are also needed for reimbursement in all indications apart from a previous stroke, TIA or systemic embolism: ° Chronic warfarin treatment is not properly controlled in the therapeutic range INR 2-3, and 2 out of 6 INR values are out of this range, or ° During the first 3 months of warfarin treatment, INR 2–3 is not reached, or warfarin is contraindicated
		*Post-launch*
		• Demand-side activities included local, regional and national events to discuss the reimbursed indications and the care needed to prescribe dabigatran (education)
Slovenia	August 2012	*Peri-launch*
		Reimbursement in line with the licensed indication in conjunction with a complex price: volume agreement
		*Post-launch*
		Demand-side activities included:
		• Education of all involved specialists and primary care physicians on key safety aspects/ adverse events with dabigatran • Prescribing restrictions (Enforcement) ° Only reimbursed if initiated by an internist or neurologist in line with approved indications. This also includes patients already on warfarin who have unstable anticoagulation, i.e., TTR < 65 ° Patients have to be followed in a tertiary or secondary care anticoagulation centre. Patients can be treated in primary care but only if authorized by the tertiary or secondary care centre. ° Every patient has to be registered in a database and followed by the IT anticoagulation programme ° Anticoagulation centres have to report once yearly to the tertiary centre regarding the number of patients experiencing minor and major bleeding, thromboembolic events, as well as any deaths from bleeding or thromboembolism
Spain (Coma et al., 2009; Ostazen, 2011)	November 2011	*(A) Post-launch activities (Basque Country)*
		(a) Assessment
		• General evaluation of dabigatran in the prevention of thromboembolism in patients with AF performed by the Drug Assessment and Vigilance Unit CEVIME of the Drug Directorate of the Ministry of Health of the Basque Country. This included a budget impact analysis under three scenarios. The budget impact for 2012 was estimated as 1.5% (7M€) of the total expenditure of primary care health prescriptions in the most restricted scenario. • A consensus statement (education) coordinated by the Ministry of Health, in collaboration with the Professional Societies of Family Physicians, Hospital and Primary Care Pharmacologists, Haematology and Haemotherapy, Cardiology, Internal Medicine and Neurology, resulted in the following restrictions for dabigatran: ° Existing patients previously treated with vitamin K antagonists (VKA) where there is hypersensitivity to acenocoumarol, warfarin or other coumarin, when INR levels cannot be properly monitored, with abnormal INR levels or when the INR is in the correct range but thromboembolic or haemorrhagic episodes are common ° New Patients: In patients with a history of ischemic stroke or high risk of intracranial haemorrhage – otherwise second line after VKAs • A post-marketing authorisation analysis is being performed by the Ministry of Health of the Basque Country to assess the actual budget impact of dabigatran • Distribution of the consensus document (education) among all healthcare professionals (primary and specialized care) and inclusion in the common electronic medical record educational tools (all the professionals have access to this medical record in primary and specialised care)
		(b) Engineering/ enforcement
		• Routine scrutiny of the prescribing dabigatran to ensure its rational use in the Basque Health System, according to the provisions of the Spanish Royal Decree 618/2007 of May 11th. Reimbursement/ funding is controlled by the Medical Inspection of the Ministry of Health of the Basque Country, with funding only granted for the prevention of stroke and systemic embolism in adult patients with non-valvular atrial fibrillation with one or more of the following risk factors: (a) Stroke, transient ischemic attack, or prior systemic embolism (ES); (b) Left ventricular ejection fraction <40%; (c) Symptomatic heart failure Class 2 or greater under the scale New York Heart Association (NYHA); (d) Age greater than or equal to 75 years; (e) Age greater than or equal to 65 associated with one of the following: diabetes mellitus, coronary heart disease or hypertension. In addition, meeting the above consensus criteria. This has helped to conserve its use in practice • The Ministry of Health of the Basque Country has included in its contracts with providers (primary and secondary care) quality of care indicators. This includes new drugs where there are concerns with their value versus existing gold standards, which now includes dabigatran. The list is updated annually and managed by the Basque Provinces directorates. • Prescriptions are electronically controlled by the Drug Directorate of the Ministry of Health of the Basque Country, and their standardized variability analysed.
		(c) Post-launch activities also included (education, engineering)
		• A centralized post-marketing authorisation follow-up of all patients receiving dabigatran • Data on patients prescribed dabigatran for AF are regularly being sent from pharmacists and clinical pharmacologists to the Ministry of Health Directorates. This electronic tool allows primary health care physicians to self-audit their prescribing and the region to monitor dabigatran use
		(B) *Post-launch activities (Catalonia)*
		(a) Education
		• General evaluation of dabigatran in the prevention of thromboembolism in patients with AF performed by the Catalan HTA. • A second evaluation undertaken by the DTC of the Catalan Institute of Health (CIH) resulted in a more restricted patient population, i.e., only in atrial fibrillation patients with (i) prior acenocumarol treatment and lack of control of INR values (2–3) in more than 60% of the last assessments, in spite of good adherence to treatment, (ii) patients who have difficulties to follow INR control and (iii) those with an allergy to acenocumarol • A third evaluation is currently being undertaken by the Catalan HTA to evaluate the different drugs available and their potential use for the prevention of thromboembolism • Distribution of a document describing the DTC decision to all Primary Health Care (PHC) physicians, as well as electronic notices and warnings regularly published on physician computers (100% of PHC physicians use computers). The same documents also distributed to hospital DTCs as well as Cardiology, Neurology, Internal Medicine and Haematology clinics.
		(b) Engineering
		• Catalan Health Service contracts with providers (primary and secondary care) to incorporate quality of care indicators including new drugs where there are concerns with their value versus existing gold standards. This now includes dabigatran, with the list updated annually
		(c) Economics
		• There are financial incentives for Catalan Health Service providers aimed at limiting the prescribing of new premium priced drugs with limited health gain versus current standards, with pertinent indicators included in the current range of quality of care indicators • Physicians who do not attain agreed standards do not receive the financial incentive
		Post-launch activities (education, engineering) also included:
		• A centralized follow-up of all patients prescribed dabigatran has been established in the CIH. Patients’ age, previous treatment with oral anticoagulants or antiplatelet agents, renal function and previous history of ischemic heart are monitored • Patients at risk of bleeding because of abnormal or unknown renal function, or with inadequate dabigatran prescriptions, are identified. Physicians in charge of these patients are contacted to confirm whether the indication for dabigatran conforms to CIH guidance and whether patients could be changed to acenocumarol • Data on patients using dabigatran for AF are regularly sent to PHC pharmacists and clinical pharmacologists, hospitals and CIH DTCs. This electronic tool allows primary health care physicians to self-audit, and the region to monitor dabigatran use • A qualitative prescription study is also currently being performed among PHCs in Barcelona. All patients prescribed dabigatran during the last semester of 2011 (*n *= 331) will be followed for12 months. The objective is to evaluate dabigatran’s effectiveness and adverse effects in practice.
Sweden (Godman et al., 2009c, 2012d; Holmström et al., 2009; Janusinfo, 2009; Stockholms läns landsting, 2011; Janusinfo, 2012a; Medicin & Läkemedel, 2012; östergötland, 2012; Davidson et al., 2013), SBU (Swedish Council on Health Technology Assessment, 2011)	November 2011	(I) National – *peri-launch* • SBU alert advisory report containing a statement that dabigatran seems to be no better than warfarin when applied to Swedish health care system • TLV did not introduce any prescribing restrictions when authorising reimbursement for dabigatran • Follow-up of patients in registries
		(II) Regional/ County activities
		(i) östergötland County Council
		*Pre-launch activities (educational, economics)*
		• Update of the previous report on the prevalence of atrial fibrillation In östergötland • Establishing a working party with broad representation from the departments of cardiology and internal medicine, primary health care, representatives from the warfarin polyclinics, epidemiologist and health economists associated with the Drug and Therapeutics Committee (DTC) • Scientific publication of the cost-effectiveness model for dabigatran for the prevention of stroke based on östergötland by the Centre for Medical Technology Assessment, Linköping University, in collaboration with östergötland County Council • Consensus action plan agreed 12 months before dabigatran was registered for the prevention of stroke in patients with AF • Recommendation from the DTC to classify dabigatran as a “focus-drug,” i.e., the prescribing unit will be responsible for the cost of the drug. If however patients are entered into the County Council’s quality assessment program, the cost will be borne by the County Council • Decision by the County Council to follow the recommendation of the DTC • Resources for treating patients allocated in the 2011–2012 drug budget • Communication plan implemented
		*Peri-launch/ post-launch*
		• Continuous follow-up of patients through a quality register • Cost of all prescriptions for patients not registered in quality register devolved to the prescribing unit
		(ii) Stockholm County Council
		*Pre-launch*
		• Extensive pre-launch activities including a critical evaluation for each approved indication • Key messages from these activities broadcasted both to the public and to prescribers through the DTC website as well as the Swedish Medical Journal • Appreciable number of pre-launch meetings and training sessions with all major physician groups around the key issues and concerns with dabigatran as well as its likely place in care. This included meetings of the “Wise List forum” • Production of educational folders regarding dabigatran, slide kits, published articles, and data on the Janus website as well as published information for patients • Forecasting the potential budget impact in 2011 and 2012 ahead of launch and monitoring this in practice
		*Peri-launch/ post-launch*
		• Development of a laboratory method to monitor dabigatran in plasma with LC-MS/MS technology • Currently recommending sampling in the introductory phase to build a knowledge database. This to be followed by more situation-based sampling to improve patient safety in the future • Post-launch guidance in the “Wise List” with warfarin recommended as first line treatment (education). In addition, budget incentives to physicians in out-patient care with all drugs in the “Wise List” not charged to their clinical budget in contrast with non-recommended drugs (economics)
Turkey	Not reimbursed	• Currently only reimbursed (75 mg) for prophylaxis in patients undergoing elective hip (maximum 35 days) or knee (maximum 10 days) replacement, and only reimbursed with special authorised reports from orthopaedic surgeons (initial); subsequent follow-up prescriptions only reimbursed via orthopaedic surgeons and subject to co-pay (enforcement) • 110 and 150 mg currently not reimbursed (100% co-pay)
